# A Discussion on the Relationship between Skin Lipid Metabolism and Whole-Body Glucose and Lipid Metabolism: Systematic Review

**DOI:** 10.4172/2576-1471.1000189

**Published:** 2018-10-10

**Authors:** Sabrina N Dumas, James M Ntambi

**Affiliations:** 1 Department of Nutritional Sciences, University of Wisconsin-Madison, Madison, Wisconsin, United States of America; 2 Department of Biochemistry, University of Wisconsin-Madison, Madison, Wisconsin, United States of America

**Keywords:** Skin, Adipose, Liver, Metabolism, Nutrition, Diet, Obesity

## Abstract

The obesity epidemic is a costly public health crisis that is not improving. In addition to the stigma and discomfort associated with carrying extra weight (at the expense of range of movement), obesity also goes hand-in-hand with co-morbidities like fatty liver disease, diabetes, cardiovascular disease, and increased risk of some forms of cancer. Currently there are no long-lasting treatments for obesity other than diet and exercise, which are not feasible for many populations that may not be equipped with the resources and/or support needed to lead a healthy lifestyle. Although there have been some pharmacological breakthroughs for treating obesity, each FDA-approved drug comes with unpleasant side-effects that make adherence unlikely. As a result, alternate approaches are necessary. In this review, we outline the relationship between skin lipid metabolism and whole-body glucose and lipid metabolism. Specifically, by summarizing studies that employed mice that were genetically modified to interrupt lipid metabolism in the skin. As a result, we propose that skin might be an overlooked, but viable target for combating obesity.

## Obesity, Metabolic Syndrome and Skin

The obesity epidemic is a costly public health crisis that is not improving. In addition to the stigma and discomfort associated with carrying extra weight (at the expense of range of movement), obesity also goes hand-in-hand with co-morbidities like fatty liver disease, diabetes, cardiovascular disease, and increased risk of some forms of cancer [1]. Currently there are no long-lasting treatments for obesity other than diet and exercise, which are not feasible for many populations that may not be equipped with the resources and/or support needed to lead a healthy lifestyle [2]. Although there have been some pharmacological breakthroughs for treating obesity, each FDA-approved drug comes with unpleasant side-effects that make adherence unlikely. For example, Orlistat is a lipase inhibitor that prevents fat absorption in the gut. However, this leads to gastrointestinal tract issues; indeed, some users have reported faecal incontinence [3]. On the other hand, Naltrexone/Bupropion, an opioid agonist that is effective for treating symptoms of metabolic syndrome, is not as effective in eliciting loss of fat mass [4]. For these reasons, alternate approaches are necessary. In this review, we outline the relationship between skin lipid metabolism and whole-body glucose and lipid metabolism and propose that skin might be an overlooked but viable target for combatting obesity.

## Functions of Skin

The skin is primarily regarded as a barrier tissue that protects individuals from ultraviolet, microbial, chemical, and physical insults [5]. To protect humans from sun-damage, the skin employs the shielding power of melanocytes [6]. In the fight against infiltration of pathogenic microorganisms, the body’s innate commensal microbes, lipids, and Langerhans immune cells work together to overcome these intruders [7]; Chemically, lipids are important for waterproofing and moisturizing the skin as well as contributing to anti-microbial defences [8]. The elasticity provided by collagen and elastin-in combination with the multi-layer design of skin-helps to maintain its integrity throughout a person’s lifetime [9]. Even though environmental protection is the skin’s primary function, other important secondary functions (detailed discussions in subsequent paragraphs) include, maintaining body temperature, maintaining whole-body water homeostasis, communicating with internal organs, and excreting both aqueous and lipid-soluble toxic material [10].

## Maintaining body temperature and water homeostasis

Thermoregulation, vasodilation, vasoconstriction, and blood flow comprise the initial mechanisms used to combat disturbances in core body temperature [11]. Dilated blood vessels and increased blood flow promote heat loss, whereas constriction and decreased blood flow retain body heat [11], which manifest as sweating and shivering, respectively [11]. Water homeostasis, or trans-epidermal water loss (TEWL), is a constitutive mechanism in which water evaporates from the surface of the skin; TEWL does not involve sweat gland activity but can lead to severe dehydration if the process is out of equilibrium [12].

## Communication with internal organs

A common example of skin communication with internal organs is the recruitment of immune cells when the skin is lacerated [13]. Briefly, chemo-attractant proteins located in the skin recruits neutrophils and monocytes to the site of the wound. Neutrophils reside in the blood, whereas monocytes reside in the spleen and bone marrow [13]. Of course, this process is not specific to skin but to all tissues that experience injury. A more remarkable and skin-specific example is that the skin can communicate with the kidney and liver in order to biosynthesize the active form of vitamin D [14].

## Excretion of toxic material

The eccrine sweat glands of the skin are equivalent to one kidney by weight, contributing to the elimination of water-soluble toxins such as heavy metals and drugs [15]. Interestingly, some substances, such as excess nicotinamide, are preferentially secreted in sweat rather than urine [16]. Secretion of sebaceous lipids has been suggested to play a role in whole-body lipid clearance in response to diet. This is because low-calorie diets lead to low levels of sebum lipids, whereas high-fat diets lead to high lipid levels in sebum [17,18]. Analysis of the sebum content on the surface of skin after high-fat feeding indicated increased triglyceride and cholesterol ester levels which is characteristic of blood lipids used to diagnose dyslipidemia [19]. Furthermore, isotretinoin, a potent anti-acne drug that remarkably decreases sebum production, leads to significantly increased blood lipids [20]. Curiously, a retrospective study of more than 11,000 men found that those who suffered from acne in their youth had significantly decreased mortality from coronary heart disease [21]. Taken together, this suggests that lipid substances excreted from the face might have long-term whole-body effects that are protective against developing lipid-related diseases such as coronary heart disease.

Even though it might be clear that the function of skin extends beyond acting as a barrier tissue, there is still some resistance to the notion that skin health can translate into whole-body health. Fortunately, researchers have created a variety of mouse models with changes in the skin-specifically in enzymes of lipid metabolism-that develop changes in whole-body metabolic function.

## The Structure of Skin

**Figure 1:**
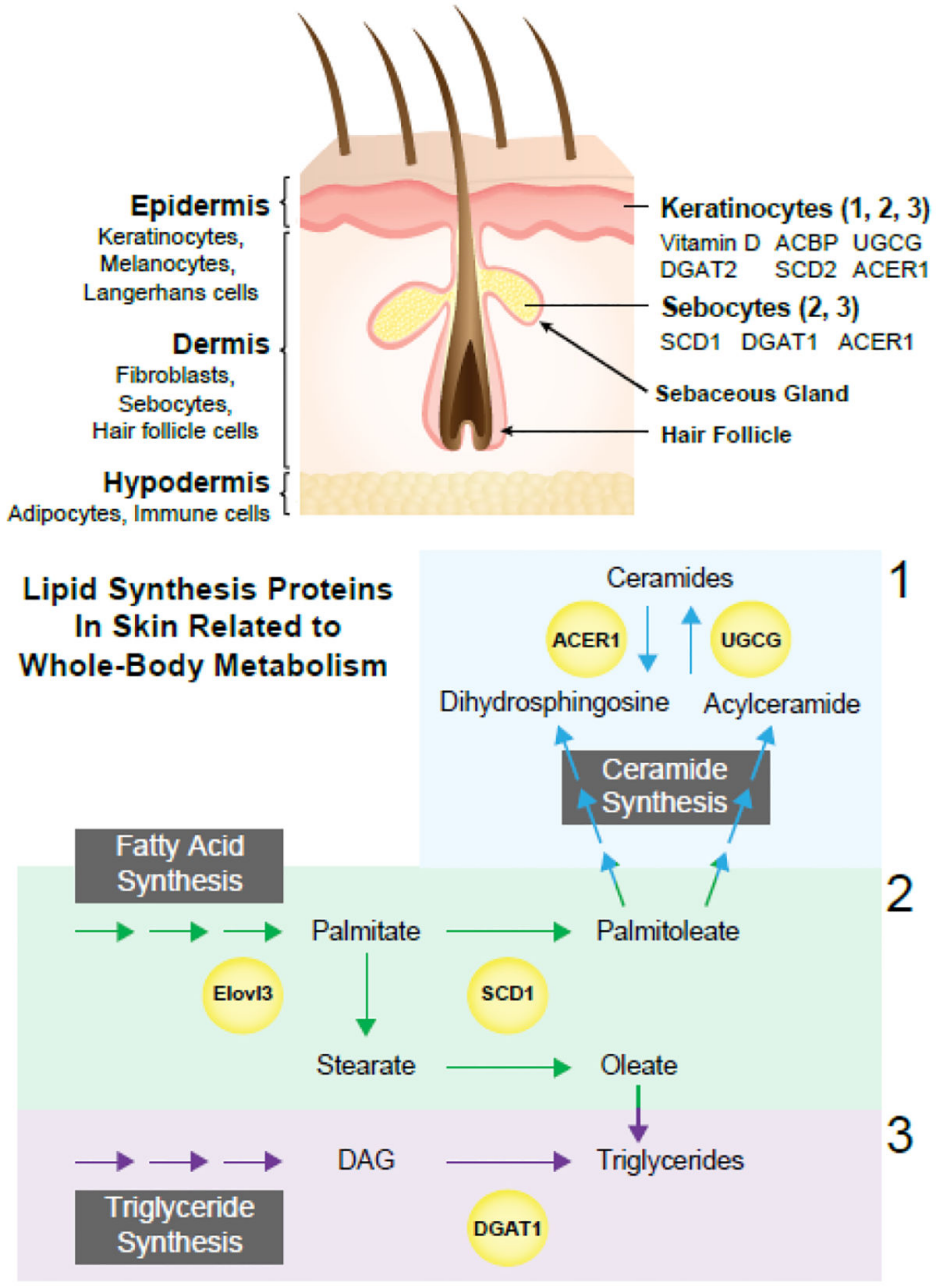
The skin is divided into three main layers, the epidermis, dermis and hypodermis with the indicated predominant cell types residing within the layers. Lipid synthesis pathways in skin; Ceramide, fatty acid and triglyceride synthesis pathways are present in either keratinocyes, sebocytes or both cell types as indicated by numbers.

A review of skin lipogenesis and metabolism necessitates a brief discussion of skin structure first. The skin is made up of 3 major layers:1) The epidermis; 2) The dermis; 3) The hypodermis [22]. The epidermis is the outer layer that is visible to the eye and is mostly made up of keratinocytes. This first layer of skin can be divided into 4 further layers: 1) The stratum basale; 2) The stratum spinosum; 3) The stratum granulosum; 4) The stratum corneum. The stratum basale is the first layer of the epidermis, which is in contact with the dermis and is the source of proliferating keratinocytes that mature towards the skin surface [22]. The stratum spinosum functions to anchor the stratum basale and stratum spinosum to each other by producing cytoskeletal proteins; and also initiates the maturation process of keratinocytes [22]. The stratum granulosum produces proteins of the corneocyte (or fully matured keratinocyte), lipids, and crosslinking enzymes that move upward to the surface [22]. The stratum corneum is the final layer that is made up of corneocytes, lipids, and cross-linked proteins and is the final, indispensable component of the skin’s function as a barrier tissue [22]. The dermis makes up the majority (90%) of the skin; it contains the hair follicles and sebaceous glands, which are collectively called the pilosebaceous unit [22,23]. The dermis is mostly made up of fibroblasts, which constitute a heterogeneous population of cells that secrete collagen, elastin, and hyaluronic acid [22]. Finally, the hypodermis (also known as the subcutaneous fat layer) anchors the dermis to the body. This highly vascularized layer is made up of white adipocytes, fibroblasts, and macrophages [22,23]. Figure 1 shows overview of skin structure.

## Lipid Species and Lipogenesis of the Skin

The stratum corneum (SC) is made up of terminally differentiated keratinocytes (called corneocytes) that are embedded in a lipid mixture of ceramides, cholesterol, and free fatty acids in a 1:1:1 molar ratio [22,24]. Since the SC makes up the visible horny layer of the skin, SC lipids also represent a portion of surface lipids. However, the larger portion is made up of sebum arising from sebaceous glands in the dermis [25]. The sebaceous gland is made up of immature and mature sebocytes that eventually undergo holocrine disruption to release its contents onto the surface of the skin [25]. The lipid components of human sebum are as follows: glycerides (30%−50%), free fatty acids (15%−30%), wax esters (26%−30%), squalene (12%−20%), cholesterol esters (3%−6%) and free cholesterol (1.5%−2.5%). It is important to note that sebum composition varies among mammalian species. Both keratinocytes and sebocytes express the full complement of *de novo* lipogenesis (DNL) enzymes [25–27]. In keratinocytes, disruption of the skin barrier elicits increased DNL [27]; this increased DNL is observed in the skin of burn patients, premature babies, and following repeated tape-stripping of the epidermis [28]. Sebocytes, similar to adipocytes, are obligate lipid producing cells, except that sebocytes produce lipids that are secreted by holocrine disruption, whereas adipocytes produce lipids for storage as energy reserves in subcutaneous, visceral, and/or epididymal white adipose depots [25,29]. Therefore, sebocytes are capable of *de novo* lipid synthesis, which sharply increases during puberty [25]. Even though both keratinocytes and sebocytes produce their own lipids, they both can also actively take up lipids from circulation. For example, essential fatty acids are found in large quantities in both skin cell types [30].

## Skin Lipogenesis and Whole-Body Metabolic Health

Vitamin D is perhaps the archetypal example of the importance of skin metabolism in whole-body metabolism. Even though it is not directly involved with lipogenesis, vitamin D is derived from a cholesterol precursor that is the product of sterol biosynthesis. Ultraviolet (UVB) rays of the sun hit the surface of the skin, where a cholesterol precursor called 7-dehydrocholesterol is converted into pre-vitamin D3 [14]. Pre-D3 must then travel to the liver to be converted into 25-hydroxyvitamin D3 (25 (OH)-D3), which is another intermediate metabolite of vitamin D synthesis [10]. Finally, (25 (OH)-D3) must travel to the kidneys, where the active form of vitamin D (1, 25-dihydroxyvitamin D3 (1, 25 (OH) 2D)) is produced [14]. Vitamin D is not only important for aiding calcium absorption in the gut, preventing the development of bone diseases such as rickets and osteomalacia, but it is also implicated in protection from prostate, breast, and colon cancer [14,31]. There is even data to support the notion that vitamin D is beneficial for type 1 diabetics because it helps to maintain β-cell function [31].

Stearoyl-CoA Desaturase 1 (SCD1), a microsomal enzyme involved in the de novo synthesis of monounsaturated fatty acids, is a Δ−9 desaturase enzyme. SCD1 adds a double bond at the 9^th^ carbon from the carboxylic acid (or delta) end of saturated fatty acids 16:0 (palmitate) and 18:0 (stearate) to make 16:1n7 (palmitoleate) and 18:1n9 (oleate), respectively [32]. SCD1 can desaturate both *de novo* synthesized and diet-derived saturated fatty acids [32]. It is ubiquitously expressed, but levels are especially high in metabolic tissues such as adipose tissue, liver, muscle, brain, and skin [32].

The SCD1 isoform is the most relevant to skin and whole-body metabolism, but other isoforms do exist in both mice and humans. Briefly, mice express isoforms 1 through 4 and humans express SCD1 and SCD5. In mice, SCD2 expression is restricted to the brain, and plays a role in embryonic development [32]. SCD3 is expressed in both the Harderian and preputial glands as well as in sebocytes [32]. And SCD4 expression is restricted to the heart [32]. In humans, SCD5 is expressed largely in the pancreas and brain [32]. In the mouse skin, SCD1 is most highly expressed in sebocytes because of its role in producing sebum-a lipid substance that incorporates oleate into many of its lipid species, including cholesterol esters, wax esters, triglycerides, diglycerides, and free fatty acids [33]. Whole-body SCD1-deficient mice are protected from diet-induced obesity and hepatic steatosis; they are also insulin sensitive and glucose tolerant [33]. Importantly, skin-specific SCD1 deficient (SKO) mice recapitulate these phenotypes and even display additional metabolic benefits, such as low blood cholesterol, which might be cardioprotective [33]. Furthermore, fatty acid oxidative genes were increased in brown and white adipose tissues, skeletal muscle, and liver, whereas lipogenic genes were either decreased or unchanged [33]. There are conflicting findings in the current literature as to whether or not loss of SCD1 in the skin increases TEWL. Briefly, two different types of spontaneous whole-body deletions of the SCD1 gene in mice-called asebia and asebia2-demonstrated different TEWL phenotypes [34]; In the asebia mouse, TEWL is not compromised because of adequate compensatory changes in skin lipids; however, in the asebia2 mouse model, compensatory changes are inadequate and TEWL is significantly increased compared to asebia mice [34]. It is believed that the difference might be attributable to mouse background effects and/or allelic effects [34]. Skin-specific SCD1 knock-out mice have not yet been tested for TEWL.

Diacylglycerol O-acyltransferases (DGAT1) is a multifunctional enzyme responsible for catalyzing several esterification reactions to make diacylglycerols, retinyl esters, and wax esters [35]. However, DGAT is most famously known for catalyzing the last step in triglyceride synthesis: converting DAG to TAG [35]. DGATs are highly expressed in lipogenic tissues such as white adipose tissue depots, small intestines, liver, and mammary glands [35]. To date, no skin-specific DGAT1 knock-out mice have been generated. However, whole-body DGAT1 deficiency leads to cutaneous phenotypes similar to SKO mice: that is, alopecia and sebaceous gland atrophy [35]. Similar to SKO mice, pups are born with fur and then lose their fur with age. Curiously, these phenotypes were reversed when DGAT1−/−mice were crossed with leptin-deficient ob/ob mice [35]. The role of leptin is unclear, but it is yet another example of skin communication with systemic tissues.

Acyl-CoA-binding protein (ACBP) is a small soluble protein that mediates the transport of long-chain acyl-CoA esters to their respective anabolic systems [36]. It also plays an important role in cytosolic lipid pool formation and is believed to act as a shield that prevents the long acyl chains from enzymatic degradation [36]. It is ubiquitously and highly expressed within the pilosebaceous unit of the skin [37]. Skin-specific ACBP knock-out mice, similar to SKO mice, experience hair loss, and dry scaly skin; they also exhibit altered lipid metabolism in the liver and white adipose tissues [37]. These mice develop fatty livers with increased levels of triglycerides and cholesterol esters; further, they exhibit increased lipolysis in white adipose tissue [37]. Interestingly, when ACBP mice are covered in Vaseline or an inert liquid latex solution, the liver and white adipose tissue phenotypes are rescued [37].

Fatty acid elongase 3 (ELOVL3) is an ER-resident protein that lengthens the acyl chain of long-chain fatty acids using malonyl-CoA as a substrate. ELOVL3 specifically elongates fatty acids ranging from C16 to C22 [38]. There are 7 ELOVL isoforms (ELOVL 1–7), each with different substrate specificities; however, only ELOVL3 deficiency has been shown to produce a skin phenotype that affects whole-body metabolism [39]. ELOVL3 is highly expressed in the sebaceous glands and epithelial cells of the hair follicles, where its elongated fatty acid products are used as substrates for ceramide synthesis. This is surprising, given that sebum is not known for high levels of ceramide content [25]. Nevertheless, without ELOVL3, mice develop alopecia and eczema and experience increased TEWL [39]. Similar to SKO mice, ELOVL3 mice are hypermetabolic and are resistant to diet-induced obesity [40]. However, these observations were made with whole-body ELOVL3 knock-out mice, so contributions from other tissues cannot be ruled out [40].

Alkaline ceramidase 1 (ACER1) is a ceramidase that maintains the levels of ceramides in the skin, thereby maintaining barrier and thermoregulatory function [41]. ACER1 is highly expressed in the keratinocytes of the epidermis and in the sebaceous glands of the dermis, another curious finding since sebum does not contain detectable amounts of ceramides [25,41]. Similar to SKO mice, ACER1 knock-out mice are hypermetabolic, hyperphagic, and lean; they also exhibit increased TEWL [41]. Although ACER1 is not expressed in white or brown adipose tissue, ACER1-deficient mice have increased levels of UCP1 in brown adipose tissue and increased beiging in white adipose tissue [41]. These strongly suggests-again-that internal organs can communicate with and respond to changes in the skin in a way that leads to remarkable changes in whole-body metabolism.

## Fatal Phenotypes

Since the primary function of skin is to protect bodily organs and tissues from external threats and dehydration, it is unsurprising that deletion of genes involved with maintaining the lipid barrier are either embryonically lethal or lead to premature death. For example, skin-specific deletion of glucosyl-ceramide synthase (UGCG) in mice leads to negligible levels of glucosylceramides in keratinocytes [42]. As a result, TEWL is sharply increased and pups die soon after birth [42]. Similarly, loss of ELOVL1 and ELOVL4 both lead to decreased levels of total ceramides and premature death [43]. More severely, DGAT2, which esterifies linoleic acid to triglycerides and acylceramides, leads to still-born pups [44]. Curiously, knock-out of SCD2 is 70% lethal; the remaining 30% that do not die prematurely display decreased skin triglycerides and ceramides and increased water loss [45]. It is worth noting that the lethal and more severe phenotypes disrupt lipogenesis in keratinocytes, while the less severe and sometimes metabolically favorable phenotypes disrupt lipogenesis in sebocytes. This correlation might be important should the skin ever become a potential target in the treatment of metabolic syndrome and obesity. Figure 1 represents lipid synthesis pathways in the skin.

## Subcutaneous Adipose Tissue

Because subcutaneous white adipose tissue is juxtaposed to skin, it may play a role in whole-body communication of skin-derived factors. Subcutaneous fat is considered the safest depot to store fat because the white adipocytes of subcutaneous fat have greater capacity to “beige” compared to visceral or epididymal adipose depots [46]. As a result, subcutaneous fat can contribute to increased energy expenditure and decreased risk of developing insulin resistance and diabetes [46]. However, if subcutaneous white adipose tissue is exposed to inflammatory stimuli, its capacity to beige and respond to insulin is decreased [47]. Consequently, patients with psoriasis are at greater risk for developing type 2 diabetes [48]. And type 1 diabetics who also develop psoriasis have reported lowering their insulin dose after starting treatment for psoriasis [49]. Furthermore, there is evidence that miRNAs secreted from inflamed psoriatic lesions lead to impairment in cholesterol efflux of the subcutaneous tissue directly below [50].

## A Non-traditional Role of Skin: Bile Acid Synthesis?

**Figure 2:**
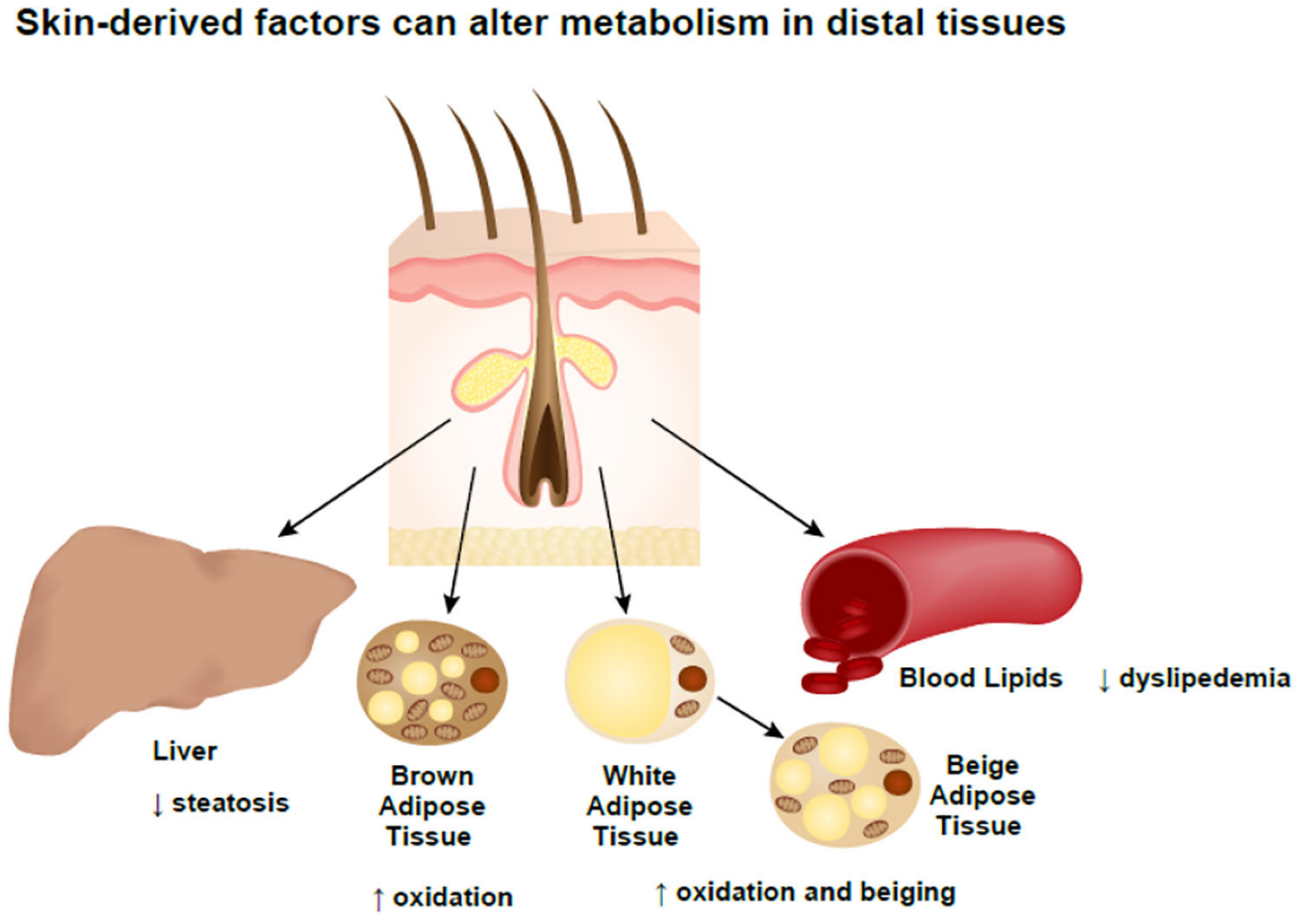
Changes in lipid synthesis pathways in skin have been linked to metabolic changes in distal tissues. Examples include decreased hepatic steatosis, increased oxidation of white and brown adipose tissue, beiging of white adipose tissue and decreased levels of blood lipids.

Recently, our lab identified a potential role for bile acids in the lean and metabolically healthy phenotype of skin-specific SCD1-deficient (SKO) mice [51]. Briefly, SKO mice have increased levels of free cholesterol on the surface of their skin [33]. Because free cholesterol is cytotoxic, we hypothesized that in addition to being effluxed from within cells, cholesterol might also be converted into a safer compound [52]. Cholesterol is a precursor for many molecules including vitamin D, steroids, oxysterols, and bile acids; we focused on bile acids because increased signaling through a bile acid-specific receptor, Takeda-G-protein-receptor-5 (TGR5), leads to similar metabolic improvements seen in SKO mice [53]. We demonstrated that SKO mice do indeed have increased levels of total bile acids in plasma, as well as the bile acid tauro-β-muricholic acid (Tβ-MCA), which is a biomarker for extrahepatic bile acid synthesis and metabolic health in mice [51,54,55]. Furthermore, a rate-limiting enzyme of extrahepatic bile acid synthesis, oxysterol 7α-hydroxylase (Cyp7b1), was significantly elevated in the skin of SKO mice [51,56]. Importantly, however, bile acid synthesis enzymes were unchanged in their livers [51]. These findings were surprising on two levels: 1) Bile acid synthesis can be initiated outside of the liver and contribute to changes in the plasma bile acid pool; 2) Skin might be a tissue capable of completely synthesizing bile acids or, at the very least, their oxysterol intermediates that must then travel to the liver for complete synthesis-a process that is not unlike vitamin D synthesis. Figure 2 shows an overview of tissues reported to respond to changes in lipid synthesis pathways of the skin.

## Challenges and Future Directions

In this review, we discussed how changes in lipid metabolism specifically in skin can lead to a variety of favorable systemic metabolic changes in several mouse models where lipogenesis genes were deleted. On the flip-side, changes that lead to defects in skin’s barrier function can lead to death due to dehydration. However, there is the example of the asebia mouse where SCD1 is deleted without compromising skin barrier function. Because deletion of SCD1 leads to a favorable metabolic phenotype, perhaps it is time to explore clinical applications where a topical application is developed and tested in a population that has an unmet therapeutic need.

An example that comes to mind is homozygous familial hypercholesterolemia (hFH) where patients demonstrate blood cholesterol up to 2000 mg/dL which is 10 times the upper limit of normal cholesterol levels [57,58]. Their current treatment is a combination of oral cholesterol-lowering drugs like statins and or bile acid resins. Some patients even have to undergo LDL aphresis where blood is cleaned and returned to the patient-a process that is similar to dialysis [59]. Skin-specific SCD1 deficient mice demonstrate low levels of blood cholesterol and high levels of cholesterol on the surface of their skin [33,51]. It is therefore an interesting idea that one might be able to modify skin cholesterol efflux to become a cholesterol sink to help clear blood cholesterol for persons with hFH. If this undertaking is successful, then we might be able to modify the SCD1 topical application to address metabolic syndrome and obesity.

Because disrupting lipid synthesis in skin will inevitably lead to dry skin, skin care measures must be taken to prevent the development of dermatological disorders. Even though, there is no guidance to be gleaned from current attempts to use skin to combat metabolic disorders in a clinical setting, there might be hints available in the treatment of skin of the elderly. Aged skin is notorious for developing severe dryness and as a result, skin barrier defects [60]. Consequently, there have been several products developed to keep aged skin healthy. Some of these products are humectants, emollients and occlusives [60]. Humectants such as urea, lactic acid and glycerin are known to decrease TEWL and skin dryness as well as increase stratum corneum hydration [60]. Emollients such as octyl octanoate or octyl stearate fill in the rough spaces of corneocytes leading to improved smoothness [60]. And occlusive like petrolatum trap water in the skin and prevent TEWL [60]. If a topical anti-obesity drug were to be developed that incorporates the features of an effective moisturizer that can keep the skin barrier function intact, then there would be greater potential for patient adherence, thereby increasing the chances of being a successful topical drug.

## Conclusion

The skin might be an as yet discovered resource to combat metabolic syndrome, dyslipidemia and obesity. There are mouse models that clearly show a causal relationship between cutaneous changes and systemic changes in metabolism. And there are human examples where a skin disease such as psoriasis is correlated with the development and or worsening of type 1 and 2 diabetes. Even a common adolescent skin disease, such as acne has implications for the development of coronary heart disease with ageing. Because of this wealth of information, it is my hope that we can begin to translate these findings into the clinic and develop alternative therapeutic interventions that can protect people from the diseases of metabolic syndrome and obesity.
